# Real-time micro-scale temperature imaging at low cost based on fluorescent intensity ratio

**DOI:** 10.1038/srep41311

**Published:** 2017-02-01

**Authors:** Jianghao Xiong, Mingshu Zhao, Xiaotian Han, Zhongmin Cao, Xiantao Wei, Yonghu Chen, Changkui Duan, Min Yin

**Affiliations:** 1 Key Laboratory of Strongly-Coupled Quantum Matter Physics, Chinese Academy of Sciences, School of Physical Sciences, University of Science and Technology of China, No. 96 Jinzhai Road, Hefei, Anhui Province, 230026, P. R. China

## Abstract

Real-time temperature imaging with high spatial resolution has been a challenging task but also one with wide potential applications. To achieve this task, temperature sensor is critical. Fluorescent materials stand out to be promising candidates due to their quick response and strong temperature dependence. However, former reported temperature imaging techniques with fluorescent materials are mainly based on point by point scanning, which cannot fulfill the requirement of real-time monitoring. Based on fluorescent intensity ratio (FIR) of two emission bands of SrB_4_O_7_:Sm^2+^, whose spatial distributions were simultaneously recorded by two cameras with special filters separately, real-time temperature imaging with high spatial resolution has been realized with low cost. The temperature resolution can reach about 2 °C in the temperature range from 120 to 280 °C; the spatial resolution is about 2.4 μm and the imaging time is as fast as one second. Adopting this system, we observed the dynamic change of a micro-scale thermal distribution on a printed circuit board (PCB). Different applications and better performance could also be achieved on this system with appropriate fluorescent materials and high sensitive CCD detectors according to the experimental environment.

Temperature is an essential parameter in both scientific research and industrial production. Spatial thermal distribution and its evolution over time are very important information in many fields, such as micro/nano-electronics, integrated photonics, biomedicine and material science^1^,^2^,^3^,^4^,^5^,^6^. In biomedicine, for instance, with the aid of high resolution real-time temperature monitoring, collateral damage could be kept at minimum in the thermal treatment of malignant tumors^6^,^7^. In micro/nano-electronics, real-time temperature imaging with high spatial resolution would also be a powerful tool to search for hot spots in integrated circuits during operation, which is demanded for design modification^2^,^8^,^9^.

In applications mentioned above, temperature imaging is often desired when we need to obtain a spatial thermal distribution. Although many types of thermal sensor have been used for temperature imaging, such as electrical thermal sensor and fluorescent thermal sensor, they can only measure local temperature of a micro/nano-meter spot in their responses time^6^,^8^,^9^,^10^,^11^,^12^,^13^. Thus in order to obtain the spatial temperature distribution in a larger scale, the sensor must be moved by mechanical device to achieve a point-by-point scanning. But the scanning process results in a very low overall imaging speed, making it impossible to monitor dynamic change of the thermal distribution. One candidate solution to this problem is two-dimensional array of micro-thermocouples, but its applications are still limited by a lot of considerable difficulties in the microfabrication procedure^14^.

A more effective real-time temperature imaging technique is infrared thermometry, based on calibration of the black-body radiation emitted from the object. While this method can obtain spatial temperature distribution in real time, its spatial resolution is not better than 10 μm, which is inherently restricted by the use of radiation with long wavelength^15^,^16^,^17^.

In order to achieve real-time temperature imaging with a better spatial resolution, we design a system based on optical imaging and use fluorescence intensity ratio (FIR) as the temperature dependent characteristic. Fluorescent materials have many temperature dependent characteristics, such as fluorescence intensity, fluorescence polarization anisotropy, fluorescence lifetime and FIR^1^,^2^,^3^,^6^,^8^,^9^,^12^,^13^,^18^,^19^,^20^. Among above temperature dependent characteristics, fluorescence intensity and FIR can achieve single-shot optical imaging. Nevertheless, fluorescence intensity is susceptible to external disturbances during the detection process, such as fluctuation of emitter amount, power of excitation light source and fluorescence loss. In contrast, FIR and fluorescence lifetime have high accuracy due to the ability to eliminate the mentioned disturbances. However, fluorescence lifetime cannot fulfill the requirement of real-time temperature imaging. As reported in ref. 6, the thermal imaging method based on fluorescence lifetime requires a reliable technique to record fluorescence decay curves, such as time-correlated single photon counting, which is complicated and also too time-consuming to achieve real-time temperature imaging. In contrast, FIR measurement of two emission bands is easy to perform, simply by using two cameras with desired filters and a beam-splitting operation. Therefore, FIR stands out as the characteristic qualified for both quick optical imaging and accurate measurement free of disturbances. We are thus inspired to build a system where spatially distributed intensity information of the two spectral bands are recorded and then processed to obtain the thermal distribution, which amounts to that this method can measure both the microscopic detail of a thermal system and the macroscopic distribution of the thermal system in real time.

In this work, adopting a rare earth doped fluorescent material SrB_4_O_7_:Sm^2+^ with high temperature sensitivity as the temperature probe, we have developed a real-time micro-scale temperature imaging system based on the FIR of two emission bands, whose spatial distributions were simultaneously recorded by two cameras with special filters separately. The relation between the FIR and temperature was calibrated, and the uncertainty of the measured temperature was evaluated. In order to test the performance of the system, we observed the stable state and dynamic change of a micro-scale thermal distribution on an operating PCB.

## Materials and Methods

### Fluorescent Material

The fluorescence of SrB_4_O_7_:Sm^2+^ was reported to be strongly temperature dependent^21^. The intensity of the emission originating from the electric dipole transition of Sm^2+^ between energy levels of 4f^5^5d^1^ and 4f^6^ configuration increases with temperature, while that originating from the inner transition from ^5^D_0_ in 4f^6^ configuration decreases with temperature, which results in a dramatic temperature dependence of FIR. SrB_4_O_7_:Sm^2+^ can be excited with ultraviolet light around 365 nm with high luminescence efficiency, so the possible laser induced heating can be avoided, which leads to less disturbance to the original temperature field. For comparison, the heating effect is usually very serious for upconversion materials excited with near infrared lasers^20^. The fluorescent material of SrB_4_O_7_:Sm^2+^ also has excellent thermal and chemical stability, and thus can be used both at temperature as high as 500 °C and in harsh environment.

SrB_4_O_7_: 5% Sm^2+^, the fluorescent material we used in this experiment, was synthesized by solid-state reaction method. In the process of preparation, SrCO_3_, H_3_BO_3_ and Sm_2_O_3_ were used as starting materials and weighed in proper stoichiometric ratio. After grinded in an agate mortar, the powder was preheated to 750 °C for 5 h. To obtain the final product, the obtained mixture was then finely powdered and heated up to 850 °C under reducing atmosphere for 10 h. The crystal phase and the mean crystalline size of the sample were identified via X-ray diffraction measurement (MAC Science co. Ltd. Mxp18.AHF, Tokyo, Japan), with nickel-filter Cu K_α_ radiation in the range of 2θ = 10°–70°. The grain size of the sample is estimated to be about hundreds of nanometers with Scherrer formula, and has important influence on the heat transfer and the spatial resolution in the experiment. If the grain size is too small, the heat transfer from the bottom surface to top surface of the SrB_4_O_7_:Sm^2+^ layer will be very slow. However, if the grain size is too big, the spatial resolution of the temperature sensing will be restricted. So, the grain size of about hundreds of nanometers is appropriate for our experiment condition.

**Figure 1:**
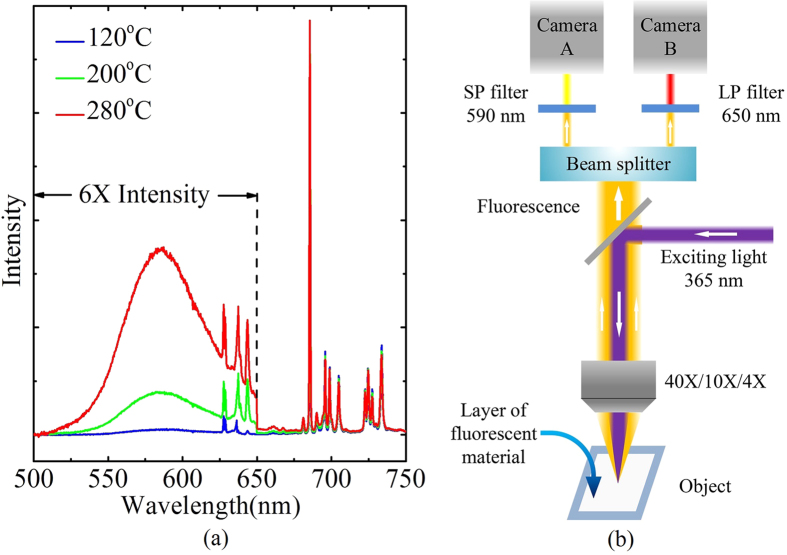
System setup. (**a**) The photoluminescence spectra of the fluorescent material excited with 365 nm ultraviolet light at the temperature of 120, 200 and 280 °C. (**b**) Sketch of the experimental setup.

Figure 1a shows the photoluminescence spectra of the fluorescent material excited with 365 nm ultraviolet light at the temperature of 120, 200 and 280 °C. Sensing signal in our experiment is FIR of the emission band from 5d (marked as *I*_A_) centered at around 585 nm to the emission peaks from ^5^D_0_ (marked as *I*_B_) located at around 684 nm. When thermal equilibrium is reached, FIR should conform to the Boltzmann law:


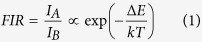


Where ∆*E* represents the effective energy separation of the emission levels, *k* is Boltzmann constant and *T* is absolute temperature.

### Experimental Setup

The whole system in our experiment is built on a fluorescent microscope. The object to be measured was placed on the platform of the microscope. The fluorescent material, temperature probe in our system, was evenly and directly pressed onto the surface of the object, with the thickness of about 50 μm. This direct contact between the object and the temperature probe can prevent contact-related artefacts reported in scanning thermal microscopy^22^. Another advantage of this method is that it permits real-time monitoring of the temperature distribution because each point in the temperature field is covered with temperature probe and thus can be observed simultaneously. The fluorescent material was excited with the 365 nm light filtered from a 100 W mercury lamp. The fluorescence emitted from the material was then equally split into two beams, separately passing through corresponding filters, a short-wave pass (SP) filter at 590 nm and a long-wave pass (LP) filter at 650 nm. Then fluorescent signals in two optical paths were recorded by two 1.3 million pixels monochrome CMOS cameras with the pixel size of 5.2 μm × 5.2 μm, followed by a further image processing to obtain the FIR images. Figure 1b shows the sketch of the experimental setup.

### Image processing

**Figure 2:**
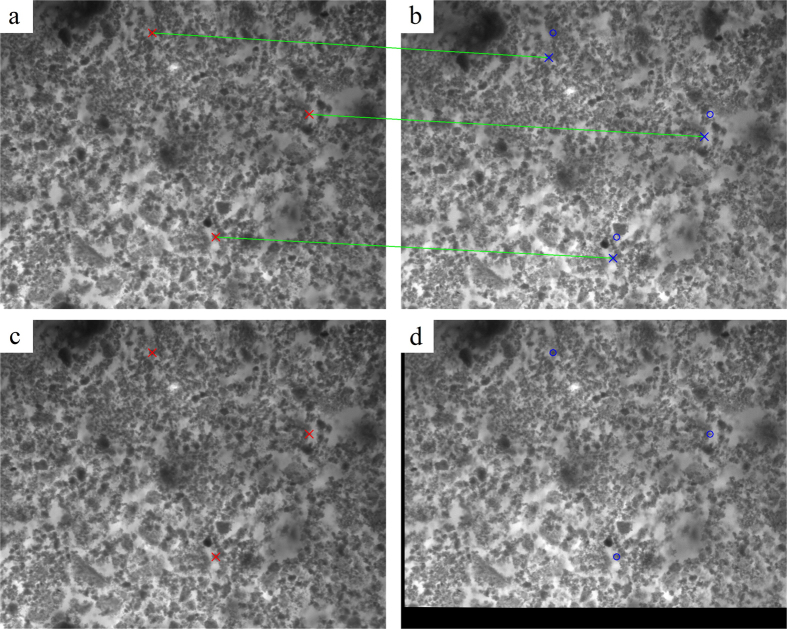
Image alignment. (**a**,**b**) Two recorded original images of fluorescent material layer deposited on the copper block, with feature points and matching result (only showing 3 matches for clarity). The crosses in two images, connected by green lines, show the matching result, while coordinates of blue circles in the right image are the same as those of red crosses in the left one. (**c**,**d**) Two images after transformation, only blue circles with the same coordinates of red crosses in the left image are shown in the right one. The black region in the right image originates from the data loss caused by the transformation.

As a result of error in optical paths, a slight difference between the two pictures simultaneously recorded by the two cameras was observed, which is inevitable and impossible to be eliminated by manual adjustment. This difference, however, can significantly compromise the accuracy, because the FIR calculation of each pixel is based on the assumption that the two corresponding pixels at different cameras reflect the physically same point. To resolve the issue, an ideal approach is to find the geometric transformation between the images taken from two cameras. If the transformation is thereafter applied into each image frame, the error in the optical paths can be eliminated. To this end, we firstly identified feature points in two images, using Scale-Invariant Feature Transform (SIFT) algorithm^23^,^24^, an effective method of identifying key points in image processing. Then according to the similarity of their features (namely distance between key-point vectors in SIFT), we matched the points, among which *N* = 30 best matches were selected. Using these matched points we deduced the transformation relation between two images in terms of rotation, translation and scaling. In this experiment, the parameters were chosen so that the standard deviation (σ) of the matched points reached the minimum, which is expressed as


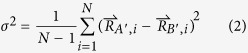


where 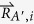
is the coordinate of the *i*_th_ feature point in the transformed image and 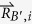 is the coordinate of the matched point in the image to be fitted. The result gives σ = 0.69 pixel. The effect of alignment is shown in Fig. 2. Noting that the precision of feature point detecting is restricted by the rounding operation after we obtain the location of the feature points. In the rounding process, feature point location’s double data type has to be converted to integer data type for image output, which yields a maximum error of 0.5 pixel for both x-axis and y-axis. Thus, the distance of two feature points can have a maximum error of 1 pixel for both x-axis and y-axis. Therefore, the deviation around 1 pixel is reasonable. After the transformation the temperature of each pixel was derived from the direct quotient of two images.

## Results and Discussion

In order to measure temperature, the relation between FIR and temperature must be calibrated first. A uniform and stable temperature field was obtained on a copper block, whose temperature was controlled by an Omron E5CC temperature controller with a type-K thermocouple and a heating tube. We assumed that the temperature field of the surface should be stable and uniform if the temperature of the copper block keeps steady for at least 10 minutes. When this requirement was reached, we simultaneously took one photo with each camera, recording the intensity of the two emission bands respectively. After the necessary transformation of photos, the FIRs of each pixel were derived from the direct quotient of two photos. Due to the defects of material, imperfection of image alignment and fluctuation of the thermodynamic system, the FIR varied in a small range. The precision, which describes the reproducibility of a measurement, can be therefore defined as the relative standard deviation of the distribution.

**Figure 3:**
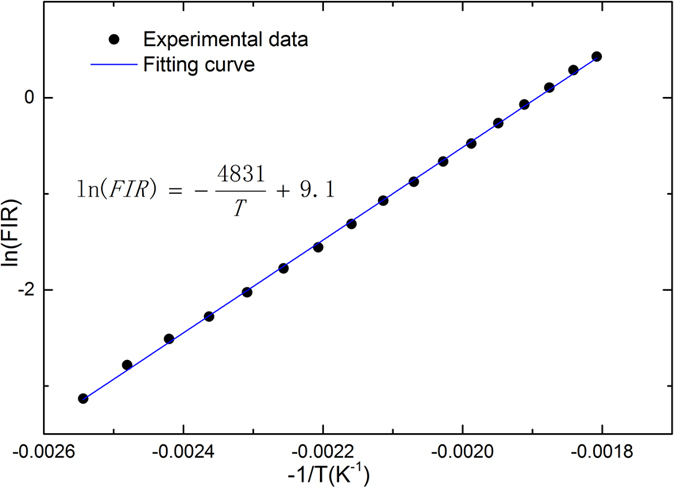
Calibration of thermal relation. Experimental data of FIR at different temperature and the fitting result with eq. (1).

The FIR images were measured in the temperature range from 120 to 280 °C with a step of 10 °C. Both procedures of temperature ascending and descending had been conducted to test the systematic stability. The average values of FIR at different temperatures were calculated from the obtained FIR images accordingly and listed in Table 1. In each measurement, we observed the value of FIR for about a minute. The fluctuation of FIR was generally below 2%, which led to temperature fluctuation below 1.2 °C. As we discussed before, the relation between FIR and temperature follows eq. (1). So the natural logarithm of FIR, ln(*FIR*), should have a linear relation with −1/*T*, and the slope will be ∆*E/k*. The experimental data follows the equation very well, as shown in Fig. 3. The ∆*E/k* was fitted as 4831 ± 29 K in the ascending procedure and 4850 ± 36 K in the descending one. Such similar results should exclude the existence of hysteresis effect in the material. Compared with the most commonly used temperature probe Er^3+^ with a ∆*E/k* of about 1000 K, the ∆*E/k* of the temperature probe SrB_4_O_7_:Sm^2+^ we used is much larger, which results in a better temperature resolution.

**Figure 4:**
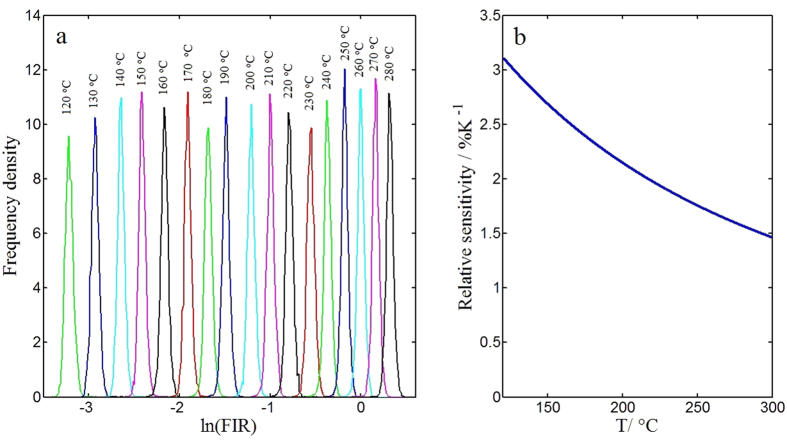
Temperature resolution analysis. (**a**) Frequency density of the natural logarithm of FIR at different temperatures from 120 °C to 280 °C with a step of 10 °C. Due to the fact that FIR increases with temperature, curves from left to right respectively correspond to temperature from 120 to 280 °C. (**b**) The variation of relative temperature sensitivity with temperature.

The frequency density distributions of ln(*FIR*) at different temperatures ranging from 120 to 280 °C are shown in Fig. 4a. With the temperature increasing, the center of ln(*FIR*) increases gradually. The standard deviations of FIR at different temperatures are calculated, and show a gradual increase with temperature as listed in Table 1. In order to evaluate the precision of the measurement, relative standard deviation of the FIR distribution was obtained via dividing the standard deviation by the average value of FIR. The relative standard deviations at different temperatures are also listed in Table 1, and are around 4% for all temperatures.

The relative sensitivity to temperature is one key parameter to determine the temperature resolution of the system, and can be defined as follow.


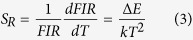


According to previous result, the value of ∆*E/k* is fitted to be 4831 K for our experimental data, so the relative temperature sensitivity can be calculated accordingly. The variation of relative temperature sensitivity with temperature in the range from 120 to 280 °C is presented in Fig. 4b. The relative temperature sensitivity can reach as high as 3.0% K^−1^ at low temperature, and is still greater than 1.0% K^−1^ at high temperature, which is better than the performance of most materials^18^,^19^,^20^,^25^.

Through the derivation of eq. (3), we evaluated the temperature resolution of our system. The temperature resolution can be related to the uncertainty of the measured temperature (*U*_T_), which is expressed as follows.


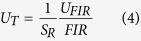


Following this formula, we derived temperature resolution of our system through substituting the uncertainty of FIR (*U*_FIR_) with the standard deviation. The obtained resolutions at different temperatures are listed in Table 1. We can see that the resolutions at different temperatures are around 2 K, which would be precise enough for application in micro/nano-electronics. The uncertainty of the FIR measurement largely determines the temperature resolution. If high sensitive imaging CCD with big dynamic range is used, the precision of the FIR measurement could be increased to better than 0.5% and the resolution could be increased to about 0.2 K accordingly.

**Figure 5:**
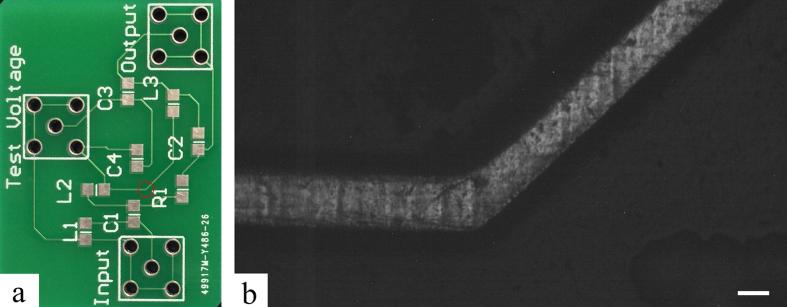
Region to be measured on PCB. (**a**) Image of the PCB in large scale with the observed area circled. (**b**) The original monochrome image of the circuit. The scale bar in the lower right corner represents 100 μm.

In order to demonstrate the performance of the system, we selected a part of 140 μm wide circuit on a PCB operating at a DC current of 5.3 A as the object to be measured, intending to observe a sharp thermal distribution resulted from the Joule heat. The full picture of the PCB was shown in Fig. 5a, where the area we observed under the microscope is marked by red circle. The grayscale image of the circuit in the circled area, recorded by cameras under the microscope, is shown in Fig. 5b. The scale bar in the lower right corner represents 100 μm. After covering the surface of the circuit with a uniform layer of the fluorescent material, the thermal distribution of the circuit was recorded.

**Figure 6:**
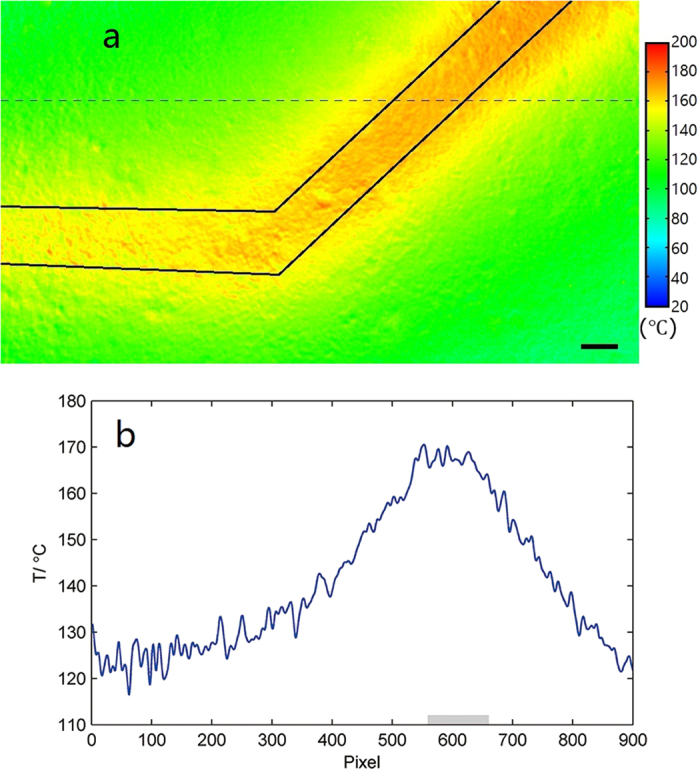
Result of measurement on PCB. (**a**) Thermal distribution of the circuit operating with a DC current of 5.3 A. The scale bar in the lower right corner represents 100 μm (outline of the circuit was drawn for clarity). (**b**) Temperature distribution along the dashed line in Fig. 6a. The gray rectangular indicates the position of the circuit.

The thermal distribution recorded after reaching thermal equilibrium is showed in Fig. 6a, where an obvious hot zone along the circuit is observed as expected. Furthermore, the part of the circuit in the top-right corner appears to have a broader hot zone compared to that in the bottom-left corner. This is believed to result from the smaller width of the circuit in the top-right corner, resulting in a greater resistance and more heat emission per unit length. In order to observe the temperature more accurately, the temperature distribution along the dashed line in Fig. 6a is drawn in Fig. 6b. We can observe an asymmetrical curve around the circuit, with the right side dropping more dramatically with the pixel. This can be explained by the asymmetrical structure of the circuit. The left side of the corner serves to conduct a bigger amount of heat to the top-left region, leading to a milder decrease on left side of the curve.

It is very tricky to discuss the spatial resolution in our system, because the specific spatial resolution of the measurement depends on the magnification of microscope. But the limiting resolution is only restricted by the diffraction limit theoretically. In the experiment, the area we try to monitor is about 1 mm × 2 mm. Thus, in order to ensure enough field of view, we had to pick 4X as the magnification, suffering a drop of spatial resolution. For a well-focused clear image obtained by camera with optical device, the spatial resolution could be estimated to be one pixel. Accompanied with the alignment error σ defined in eq. (2) for our experiment, the spatial resolution of our image could be roughly estimate as 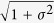 pixel, namely 2.4 μm under 4X as the optical magnification.

**Figure 7:**
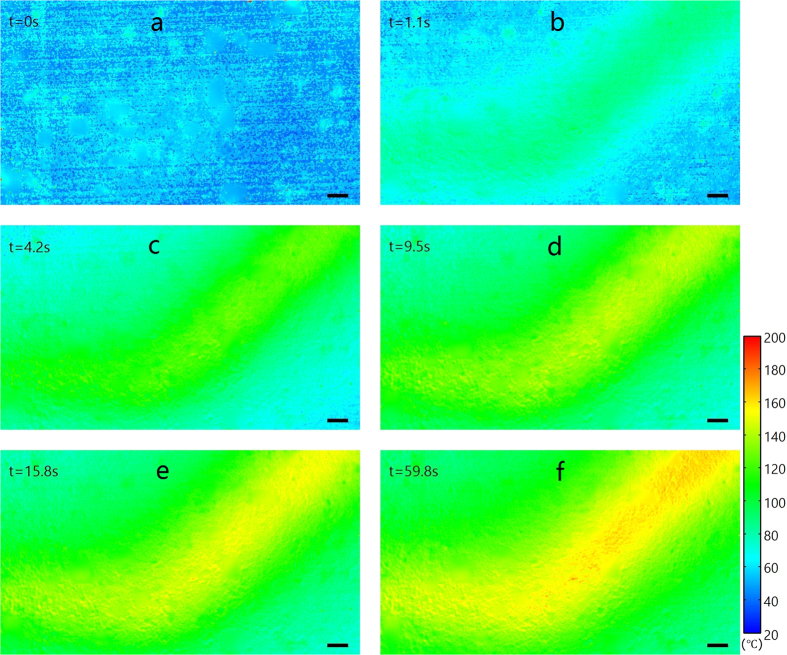
Dynamic change of temperature distribution on the circuit. The circuit was operated with a DC current of 5.3 A, where t = 0 s represents the time when the current is on. (**a**–**f**) Thermal distributions at t = 0, 1.1, 4.2, 9.5, 15.8 and 59.8 s. The scale bar in the lower right corner represents 100 μm.

The dynamic change of thermal field on the circuit operating with a DC current of 5.3 A is shown in Fig. 7, where the establishment process of the thermal field can be observed. The temporal resolution is restricted by the exposure time of the camera, which was set as 1.1 seconds in our experiment due to the low signal level of the emission band around 585 nm in the temperature range we measured. Therefore, if the requirement of temporal resolution is higher, the improvement can be easily achieved by increasing the signal with a more powerful light source or a highly sensitive imaging CCD, such as electron-multiplying CCD (EMCCD) and intensified CCD (ICCD) which has the ability to obtain a temporal resolution up to several nanoseconds.

## Conclusions

Based on the FIR of two emission bands, we built a low-cost thermal imaging system with a fluorescent microscope, two filters, and two cameras to reach the requirement of good spatial, temporal and thermal resolution for real-time micro-scale temperature imaging. The temperature of each point is derived from the intensity ratio of two pixels at the same point on two photos. The fluorescent intensity of the two pixels is simultaneously recorded by two cameras with different filters. We calibrated the relation between FIR and temperature, and evaluated the temperature resolution. Then we obtained a series of time-evolving thermal images of an operating PCB circuit, where the establishing process of a conspicuous thermal distribution around the circuit was observed. The results showed the good spatial and temporal resolution of the system. Performance of the system could easily be improved by increasing the signal with a more powerful light source or a high sensitive imaging CCD for more precise measurement, if the high cost is acceptable.

## Additional Information

**How to cite this article**: Xiong, J. *et al*. Real-time micro-scale temperature imaging at low cost based on fluorescent intensity ratio. *Sci. Rep.* **7**, 41311; doi: 10.1038/srep41311 (2017).

**Publisher's note:** Springer Nature remains neutral with regard to jurisdictional claims in published maps and institutional affiliations.

## Figures and Tables

**Table 1:** Several parameters in temperature calibration.

T (°C)	The average value of FIR	Standard deviation	Precision	Resolution of T (°C)
120	0.0436	0.0021	0.049	1.6
130	0.0618	0.0028	0.045	1.5
140	0.0811	0.0034	0.042	1.5
150	0.1024	0.0042	0.041	1.5
160	0.1210	0.0045	0.037	1.4
170	0.1690	0.0059	0.035	1.4
180	0.2110	0.0073	0.035	1.4
190	0.269	0.010	0.037	1.6
200	0.343	0.013	0.038	1.7
210	0.417	0.015	0.035	1.6
220	0.515	0.018	0.035	1.7
230	0.621	0.023	0.037	1.9
240	0.765	0.029	0.038	2.0
250	0.932	0.035	0.038	2.1
260	1.111	0.042	0.038	2.1
270	1.334	0.051	0.039	2.3
280	1.537	0.058	0.038	2.3
